# Behavior of Embedded Cation-Exchange Particles in a DC Electric Field

**DOI:** 10.3390/ijms20143579

**Published:** 2019-07-22

**Authors:** Lucie Vobecká, Tomáš Belloň, Zdeněk Slouka

**Affiliations:** 1 Department of Chemical Engineering, University of Chemistry and Technology Prague, Technická 3, Prague 16628, Czech Republic; 2 New Technologies–Research Centre, University of West Bohemia, Univerzitní 8, Pilsen 30614, Czech Republic

**Keywords:** ion-exchange membranes, ion-exchange particles, heterogeneity, electrokinetics, current–voltage curves

## Abstract

Electrodialysis and electrodeionization are separation processes whose performance depends on the quality and properties of ion-exchange membranes. One of the features that largely affects these properties is heterogeneity of the membranes both on the macroscopic and microscopic level. Macroscopic heterogeneity is an intrinsic property of heterogeneous ion-exchange membranes. In these membranes, the functional ion-exchange component is dispersed in a non-conductive binder. The functional component is finely ground ion-exchange resin particles. The understanding of the effect of structure on the heterogeneous membrane properties and behavior is thus of utmost importance since it does not only affect the actual performance but also the cost and therefore competitiveness of the aforementioned separation processes. Here we study the electrokinetic behavior of cation-exchange resin particle systems with well-defined geometrical structure. This approach can be understood as a bottom up approach regarding the membrane preparation. We prepare a structured cation-exchange membrane by using its fundamental component, which is the ion exchange resin. We then perform an experimental study with four different experimental systems in which the number of used cation-exchange particles changes from 1 to 4. These systems are studied by means of basic electrochemical characterization measurements, such as measurement of current–voltage curves and direct optical observation of phenomena that occur at the interface between the ion-exchange system and the adjacent electrolyte. Our work aims at better understanding of the relation between the structure and the membrane properties and of how structure affects electrokinetic behavior of these systems.

## 1. Introduction

Directed transport of ions by ion-exchange membranes when in DC (direct current) electric field is pertinent to electromembrane separation processes such as electrodialysis and electrodeionization [1,2,3]. These membranes possess a property called ion selectivity, which stems from a combination of two important factors [4,5]. The first factor is a so-called fixed charge that can act on mobile ions in the electrolyte solutions by exerting electrostatic forces. The second factor is the very structure of the functional part of ion-exchange membranes [6]. The internal structure has to have characteristic dimensions on the nanometer scale so that the electrostatic attraction or repulsion between the fixed charges and the mobile ions can occur. The ion-exchange membranes can then be classified into cation and anion-exchange membranes, which is given by the charge of the functional group fixed in the membrane [7]. Another classification divides the membranes into two groups on the basis of their macroscopic compositions. The first group represents membranes that are viewed as homogeneous on the macroscopic level, i.e., the fixed charge is present throughout the membrane [7]. The second group is a group of so called heterogeneous membranes having macroscopic domains of different composition and internal structure and thus different functionality. Typical heterogeneous membranes consist of functional ion-exchange material (usually in the form of finely ground resin particles) and an inert binder, which houses the functional component and provides the membrane with mechanical stability [8,9]. The functional component can be viewed as homogenous. Often polymeric fibers are laid on the surface of these membranes. Each type of the membranes brings its benefits and disadvantages. While the homogenous membranes usually surpass the heterogeneous ones in the electrochemical characteristics (resistance, selectivity, etc.) [10], the heterogeneous membranes possess better mechanical and chemical stability and their production cost is lower. This fact motivates the research focusing on better understanding of the ion transport in case of heterogeneous membranes, which is reflected in their properties such as resistance or the selectivity.

The transport across ion-exchange membranes when polarized in DC electric field results in an interesting current–voltage curve [11,12,13]. In a broader sense, the current–voltage curve can be viewed as a dependence of the flux (flow) on the driving force. In the case of the ion-exchange membranes, the flux is represented by the current density (electric current carried by ions) and the driving force by the electric voltage. The current–voltage curve usually consists of three major regions, which are referred to as an underlimiting, limiting, and overlimiting one [14]. The underlimiting region occurs at low voltages when enough ions able to carry the electric current are present on either side of the membrane. However, their concentration decreases with increasing voltage on one side of the membrane, which is a result of a so-called concentration polarization phenomenon. This side of the membrane is denoted as the depletion side. This side controls the ionic current going through the system at higher voltages and thus the behavior of the whole system. The concentration polarization [15,16,17] predicts that the concentration of ions on the depletion side drops essentially to zero at certain value of polarizing voltage and that the electric current will reach a limiting value, i.e., it is not possible to achieve a higher current by increasing the voltage. The tendency of current to level off is typical for the limiting region. However, it was found out that true saturation in most cases does not occur [18] and one can observe an overlimiting current in these systems. The overlimiting current is still under scientific scrutiny and many theories have been developed to explain its appearance [19,20,21]. All theories, no matter what system they apply to, predict the appearance of new or fresh ions in the depletion region, which in other words means partial destruction of the depletion region itself. There are two major mechanisms accepted in the scientific community that can lead to the appearance of new ions in the depletion region and these are: (i) Convective mixing invoked by the conditions on the depletion side of the membrane [22,23,24] and (ii) water splitting reaction generating hydrogen and hydroxide ions [25,26,27]. The convective mixing can be driven either by the electric field itself and the resulting phenomenon is called electroconvection or by natural convection, which is associated with the generation of gradients in the density of the desalted electrolyte solution. These gradients are caused by variations in concentrations or temperatures. The appearance of the overlimiting current makes ion-exchange membrane based separation unique in the area of membrane separation. The performance of other membrane separation processes is limited by the concentration polarization [28] (such as reverse osmosis). Concentration polarization does not allow one to reach flux of the given component larger than the limiting one. This fact attracts scientific attention since if used properly in electrodialysis, the exploitation of convective mixing producing overlimiting current can lead to significant intensification of these processes [20,22]. However, today most of the electrodialysis units are operated under underlimiting current conditions since appearance of the overlimiting currents can be accompanied by processes with deleterious effect on the desalination. Extensive release of Joule’s heat or water splitting reaction can be named as examples. Thorough understanding of the overlimiting mechanism and their effect is thus necessary.

Electroconvection as the mechanism behind the overlimiting current always results from interaction of a strong electric field with a spatial mobile charge that is localized between the electrolyte solution and the ion-exchange membrane. There are a number of different theories as to why electroconvection occurs. A nice overview of the current understanding of electroconvection was given by Nikonenko et al. [20]. Today one acknowledges that the structure of the membrane plays a significant role in the development of electroconvection. It has been found that both surface profiling of homogenous ion-exchange membranes [29,30] and presence of heterogeneities in case of heterogeneous ion-exchange membranes [26,31] have profound effect on the developing electroconvection. In general, profiling or heterogeneity cause the electric field to deviate from the perpendicular direction towards the membrane, which results in a substantial tangent electric field component with strong electric force on the extended polarized layer on the membrane surface. This tangent component then produces electrokinetic flow on the membrane whose structure depends on the geometry of the system and the experimental conditions. Until now, electroconvection has been studied for given membranes or membranes with given profiling or structuring.

In this work we attempt to produce “membranes” containing spatially arranged whole cation-exchange resin particles. We study the effect of the number of particles on (i) the measured current–voltage curve (CVC) curves, (ii) electrokinetic effects developing around the particles during polarization, and (iii) the tendency of these systems to split water. The main aim of manuscript is to describe differences one can observe in these systems when producing membranes with different number of ion-exchange particles.

## 2. Results

This section is organized as follows. In the first part of the section, the individual membranes are studied experimentally with respect to the exhibited current–voltage curve (CVC) and the behavior of the electrolyte on its depletion side of the membrane. Our experimental setup allows us to detect the appearance of electroconvection when the CVC is measured and to track the main motion of the electrolyte that develops around the membranes. In the second part of this section, we test some of the systems for their susceptibility to a water splitting reaction.

### 2.1. Studied Membranes—Current–Voltage Curves

The membranes studied in this work are homemade membranes consisting of a certain number of cation-exchange resin particles. The number of particles, we could incorporate into our membranes, was limited by our requirement of the observation of all particles under microscope. For this reason, the membranes prepared for this study contained one, two, three, and four particles. The two- and three-particle membranes were made so that the particles were positioned horizontally in a line, the four-particle membrane had two rows of particles, i.e., upper row contained two particles and the bottom row also contained two particles. We determined the ion-exchange area of these membranes by using the strategy described in Section 4.3. The measured distances used in the evaluation of these surface areas are given in figures showing the result for individual studied systems.

#### 2.1.1. One-Particle System

**Figure 1:**
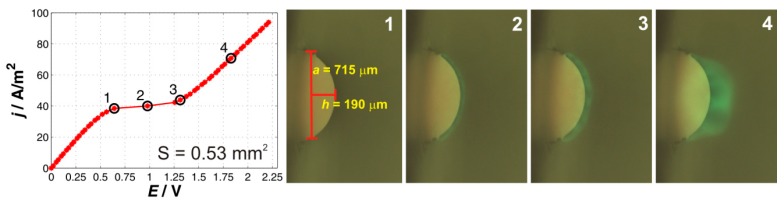
Current–voltage curve and a set of characteristic images for the one-particle membrane. The black circles on the current–voltage curve mark the current densities at which the depicted fluorescent images denoted as 1, 2, 3, and 4 were obtained.

The results for the one-particle membrane are shown in Figure 1. The Figure shows the measured current–voltage curve on the left and then a set of fluorescent images, which capture the situation on the depletion side of the membrane during polarization. Specifically, the fluorescent pictures show the situation at specific points on the CVC, which are of special interest. Point 1 shows the transition of the system from the underlimiting to the limiting region, point 2 the situation in the limiting region, point 3 during the transition to the overlimiting region, and point 4 in the fully developed overlimiting region. The same notation is also used in the description of the results for the other multi-particle membranes.

The current–voltage curve shows all three regions that are typical for the ion-exchange systems, i.e., underlimiting, limiting, and overlimiting regions. The limiting current density reached a value of 38.4 A/m^2^. By looking at the shape of the CVC, one clearly sees that the underlimiting region had the highest slope and the limiting one had the lowest slope. The values of the slopes for the underlimiting and overlimiting regions, which also indirectly provide a picture about the conductivity of the system in the given region, were 72.7 and 60.5 A/m^2^/V. The value of the overlimiting slope was smaller than that for the underlimiting region, which is consistent with the fact that the mechanism driving the overlimiting current cannot fully restore the conditions corresponding to the underlimiting region. To decipher what mechanism could be responsible for the overlimiting current, we synchronized the obtained images with the measurement of the CVC. One can see from Figure 1 that at the transition from limiting to overlimiting region, there were no visible characteristic features at the interface (point 1). However, right after transitioning into the limiting region (point 2) one can see the appearance of a fluorescent wave that went from the particle outward. In the third frame, which depicts the situation at the transition from the limiting to the overlimiting region, the fluorescent zone widened and contained an array of dark spots that were evenly distributed around the particles. On analyzing the obtained images, one will see that the dark spots are small vortices that form on the surface of the particles. These vortices grow with polarizing current and merge into a few larger ones when the membrane is fully in the overlimiting region (see Figure 1 frame 4). The obtained images clearly show that electroconvection played an important role in the appearance of the overlimiting current. These observations are consistent with our previous results [14]. The role of possible water will be discussed later. This one-particle membrane system serves as a basic system to which the others will be compared.

#### 2.1.2. Two-Particle System

**Figure 2:**
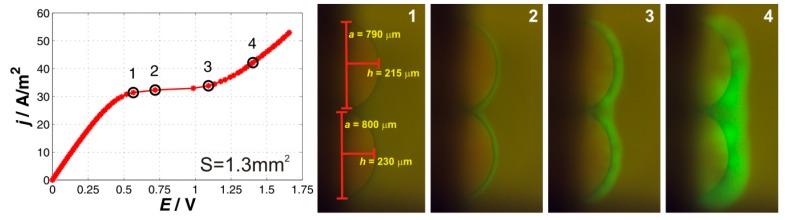
Current–voltage curve and a set of characteristic images for the two-particle membrane. The black circles on the current–voltage curve mark the current densities at which the depicted fluorescent images denoted as 1, 2, 3, and 4 were obtained.

The results for the two-particle system are shown in Figure 2. The current–voltage curve again shows three distinctive regions characteristic for the ion-exchange systems with no qualitative changes when compared to the one-particle system. The underlimiting region, which possesses the slope of 73 A/m^2^/V, transitions into the limiting one at the current density of 31.4 A/m^2^. While the slope of the underlimiting region was very similar to that of the one-particle membrane, the limiting current density was lower. This might be given by the close arrangement of the two particles. The particles share the space of depletion, thus a lower current is needed to reach the limiting conditions. The transition from the limiting to the overlimiting region was more gradual than in the previous. The slope of the overlimiting region was 43.6 A/m^2^/V, which is approximately 30 percent lower than in the case of the one-particle system. One explanation of this observation is that the ion-exchange area is larger and thus the local electric field is not that strong, which in turn means that all overlimiting processes are driven with an electric field of lower strength.

The accompanied fluorescent pictures show how the system behaves at the transition from the underlimiting to the limiting region (point 1), in the limiting region (point 2), at the transition from the limiting to the overlimiting region (point 3), and in the overlimiting region (point 4). The description of the observed behavior given for one-particle membrane is also qualitatively valid for the two-particle system. The first fluorescence appears when the system was in the limiting regions (point 2). This fluorescence emanated from around the whole ion-exchange surface and its intensity was evenly distributed. An array of dark spots appeared within the fluorescent region and grew in size with the polarizing current (point 3). One characteristic dark spot was in between the particles. These dark spots remained there until the end of polarization. In the overlimiting region, smaller vortices merged into a smaller number of the larger ones (point 4).

#### 2.1.3. Three-Particle System

**Figure 3:**
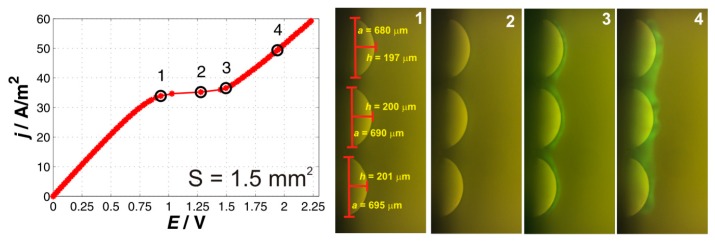
Current–voltage curve and a set of characteristic images for the three-particle membrane. The black circles on the current–voltage curve mark the current densities at which the depicted fluorescent images denoted as 1, 2, 3, and 4 were obtained.

The results for the three-particle membranes are depicted in Figure 3. The current–voltage curve shows three typical regions with no significant differences in its quality when compared to the previous cases. The slopes of the underlimiting and overlimiting regions were 41 and 33.2 A/m^2^/V, respectively, the limiting current density reached value of 34.7 A/m^2^. When compared to the previous cases, both the underlimiting and overlimiting regions showed smaller conductivity. The explanation for the overlimiting region might be given in a weaker electric field than that in the case of a larger area it will not attain such strength. The increased resistance of the underlimiting might have its origin in increased resistance on the other side of the membrane or between the membrane and the reference electrodes.

The fluorescent images again showed development of the fluorescent zone with darker spots, which grew with polarizing current. Two characteristic dark spots were again seen between the particles. One qualitative difference when compared to the previous membranes was that the first fluorescence was detected at the transition from the limiting to the overlimiting current (see point 2 and 3). The appearance was thus delayed with respect to previous cases, which points at the fact that another resistance was present in the system. This resistance caused the electric field to readjust accordingly. The fluorescent images, however, clearly showed that electroconvection played an important role and was definitely one of the mechanisms driving the overlimiting current.

#### 2.1.4. Four-Particle System

**Figure 4:**
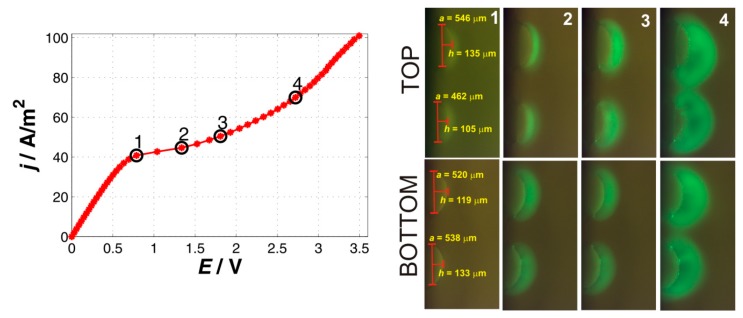
Current–voltage curve and a set of characteristic images for the four-particle membrane. The black circles on the current-voltage curve mark the current densities at which the depicted fluorescent images denoted as 1, 2, 3, and 4 were obtained.

The four-particle membrane differed from the previous membranes in that the particles are arranged in two rows (top and bottom) with each row having two particles. The fluorescent images in Figure 4 thus depict two sets of images, one for particles in the top row and the other in the bottom row. The images were obtained by selective focus on the given row of particles.

The current–voltage curve for the four-particle membrane bears a shape typical for the ion-exchange systems. The underlimiting region was characterized by the slope of 64.4 A/m^2^/V, which is very close to the slope observed for the one and two-particle systems. The transition to the limiting regions occurred at the current density of 40 A/m^2^, which is a value very close to that measured for a single particle. By inspecting the images of the four-particle membrane (especially frames for point 2 in Figure 4), one will see that the system essentially consisted of four particles that are isolated, i.e., they act as individual ion-exchange domains. This is nicely documented by the fluorescent signal (point 2 in Figure 4) that is disconnected unlike the previous cases in which the fluorescent signal was continuous along the whole particle membrane. This system can thus be considered as four non-interacting single particles connected in parallel. While the transition from the underlimiting to the limiting region was very well defined, the transition from the limiting to the overlimiting current was very slow. The slope of the overlimiting region above point 4 on the CVC was 44.6 A/m^2^/V, which is comparable to previous systems, however, this value was obtained at a much larger driving voltage around 3 V. The slope between the point 3 and 4 was much smaller. The explanation of this gradual transition from the limiting to the overlimiting region can be found in the fact that the transition from the limiting to the overlimiting region of individual particles in this system was not synchronized and occurred at different currents and voltages. This hypothetical explanation had its support in the fluorescent images (point 2 and 3) in which the upper particles seem to be behind the bottom particles in the development of the fluorescent wave with dark regions corresponding to the array of vortices. The qualitative behavior regarding the formation of the vortices and their growth was very similar to the previous systems. One specific feature, however, was the formation of very bright small dots at the particles surface (point 4). These spots look like stationary stagnant points between the individual vortices formed on the particles in which fluorescein can accumulate.

### 2.2. pH Changes at Studied Systems

In all the previous experiments, we were able to identify electroconvection that develops on the studied systems during the transitions from the limiting to the overlimiting regions of the CVCs. The other mechanism often mentioned with the appearance of the overlimiting current is the water splitting reaction. Although the mechanism of the reaction might be complex, its occurrence was manifested in the changes in pH values in the solutions adjacent to the ion-exchange system at which the aforementioned reaction took place. This is of course true in the case when no pH buffers were present in the solutions.

**Figure 5:**
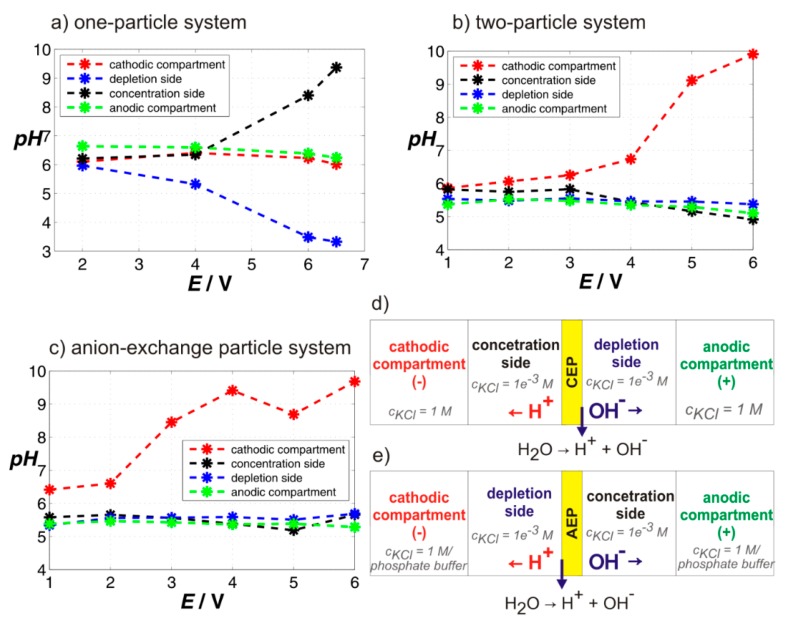
pH changes in the solutions taken from individual chambers of the cell after running chronoamperometric measurement for 5 min. The graphs (**a**) and (**b**) are for one- and two-particle membranes studied here, graph (**c**) for an anion-exchange resin particle serving as a control system. The schematics (**d**) and (**e**) depict the situation which would occur in a cation- and anion-exchange system with the proceeding water splitting reaction, respectively.

To reveal any possible contributions of water splitting reaction to the overlimiting current, we performed experiments in which various voltages were applied on the system for 5 min. After that we mixed the solutions in all chambers and measured their pH. These experiments were carried out for one- and two-particle systems and a single anion-exchange particle as a control. The results of these experiments are plotted in Figure 5a–c. Each of the graphs shows the dependence of the pH of the four solutions from different chambers on the applied voltage. As can be seen in Figure 5a,b for the one- and two-particle membranes, the pH in the chambers adjacent to the membrane did not depend on the voltage (blue curve for the depletion side, black curve for the concentration side) and was equal to the pH of the solution measured at the underlimiting voltage of 1 V (no changes expected). The same was true for the anodic compartment (green curve). The pH in the cathodic compartment (cathode) increased with increasing voltage (red curve in Figure 5a,b). This pH increase was given by a water splitting reaction that occurred on this electrode. However, the pH changes in this compartment did not affect the pH in the neighboring chamber in which the pH was constant (depletion side). The schematic in Figure 5d shows a hypothetical situation in a cation-exchange system in which the water splitting reaction would occur. The generation of H^+^ and OH^-^ ions would result in a pH decrease on the concentration side of the membrane and pH increase on the depletion side of the membrane. Moreover, this pH change would be voltage dependent. Neither was observed for the studied particles, which led to the conclusion that water splitting reaction did not occur in these systems, or its extent was very low.

The results for the anion-exchange particle are plotted in Figure 5c. In this particular case, the anion-exchange particle turned out to be very efficient in splitting water. While the pH of the solution on the depletion side decreased, the pH of the solution on the concentration side increased and the pH changes were voltage dependent. The higher voltage the larger the pH change. These pH changes were in good agreement with the schematic in Figure 5e, which showed a situation in an anion-exchange system with the proceeding water splitting reaction. The pH of the solutions in the electrode compartments did not change for any of the applied voltages (we added another electrode chamber filled with phosphate buffer to our cell to prevent observed changes in the cathodic compartments). The results obtained on the anion-exchange particle, which are known to split water, were used as a positive control regarding our experimental setup for the determination of pH changes in our cell.

## 3. Discussion

The presented study aims at gaining a better understanding of the behavior of ion-exchange systems in which geometrical complexity is increased by addition of ion-exchange domains. The study investigates four different systems that contain 1, 2, 3, or 4 cation-exchange resin particles. While the particles were arranged in a horizontal line in one-, two-, and three-particle membranes, the particles in the four-particle membrane were arranged in a square. Since these systems are difficult to fabricate, we resorted to studying the individual systems from the point of quality, i.e., what behavior these systems exhibit and suggest what might be the cause for the observed differences.

All studied systems exhibited behavior typical for ion-exchange systems, i.e., their current–voltage characteristics bear the shape typical for these systems. The accompanying recordings of the processes developing on the membranes revealed that electroconvection develops in all systems when the systems transition from the limiting to the overlimiting region. This electroconvection presents itself as an array of vortices that grows with increasing polarizing current. From the qualitative perspective, we did not observe significant differences in the produced electroconvection for systems with a different number of particles. This evidences electroconvection as one of the major mechanisms responsible for the overlimiting current. Measurement of the pH changes around the one- and two-particle membrane showed that water splitting did not occur in the studied cation-exchange systems unlike the anion-exchange system. The anion-exchange resin particle was used as a positive control. Overall the study showed that no matter how many cation-exchange particles we had in the system, they would always show the current–voltage curve typical for ion-exchange systems and the electroconvection would play a major role in the appearance of the overlimiting current.

**Figure 6:**
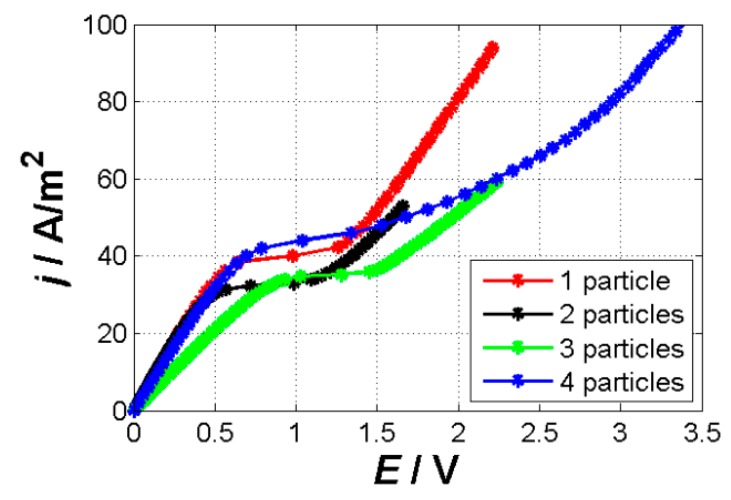
Current–voltage curves of the studied systems. The red curve represents the one-particle systems, the black one the two-particle system, the green one the three-particle system, and the blue one the four-particle system. We evaluated the slope of the underlimiting and overlimiting regions and the limiting current density, which are given in Table 1.

From the quantitative perspective, we found differences in the behavior of the four studied systems. The origin of the differences is most probably associated with the way the particles forming the membrane are positioned one to another and how much of their surface is available for the ion-exchange. Figure 6 shows the measured CVC curves for all studied systems and the Table 1 shows some of the characteristics evaluated from these CVC curves along with the surfaces of individual systems. This table documents the following observations. The limiting current density was larger for systems in which the surfaces of individual particles making up the membrane were smaller. The smaller the surface of the particles was the more it acts as a point source with a radial impact on the surrounding electrolyte. In other words the volume of the surrounding electrolyte required to be depleted to reach the limiting region was larger for smaller isolated surfaces (such as the four-particle membrane, Figure 4, point 1,2) than that for larger interacting surfaces (two-particle membrane, Figure 2, point 1,2). Larger, non-isolated particles might “share” the depletion zone and thus lower current is needed to reach depletion. The overall surface did not seem to play an essential role in evaluating the limiting current density.

These observations are consistent with experimental data of e.g., Butylskii [32] or Green et al. [33]. In [32], the authors studied systems with surface electric inhomogeneities at which they found the appearance of two transition times during the measurement of chronopotentiometric curves. The first transition time is associated with the depletion of ions at conductive domains. The value of the first transition time is strongly affected by not only normal diffusion but also tangential diffusion (diffusion from non-conductive domains to the conductive domains). The second transition time is associated with depletion of ions over the whole membrane. In this case, electroconvection developing at the interface between conductive and nonconductive regions at around the first transition time plays a significant role in ion transport to the conductive domains [34,35]. In [33] the authors studied the effect of conductive heterogeneity on systems of parallel ion-selective nanochannels. Their experimental data clearly showed that an increase in the system heterogeneity (decrease in the number of parallel nanochannels or increase in their spacing) leads to the increase in the underlimiting conductance and the limiting current.

The number of particles did not significantly affect the slope of the underlimiting region. Only the system with three particles had a significantly lower value of this parameter, which is most probably given by the additional resistance in the system introduced during fabrication. The slope of the over-limiting region did seem to be larger for systems with a smaller overall area, however there might be many other effects playing an important role in this case. Since electroconvection was established as the major mechanism for the overlimiting current, one should account for the actual geometry of the systems, the voltage and current at which the overlimiting region occurs, etc. The description of these effects requires further experimental work. 

## 4. Materials and Methods

### 4.1. Materials

The materials used in this work include: Cation-exchange resin particle Dowex^®^ 21K from Sigma-Aldrich (Prague, Czech Republic), anion-exchange particle Suquing 201 × 4 Cl. The chemicals used in this work include: KCl from Penta a.s. (Prague, Czech Republic), and fluorescein (acid free) from Fluka (ordering number: 46955).

### 4.2. Electrochemical Cell

**Figure 7:**
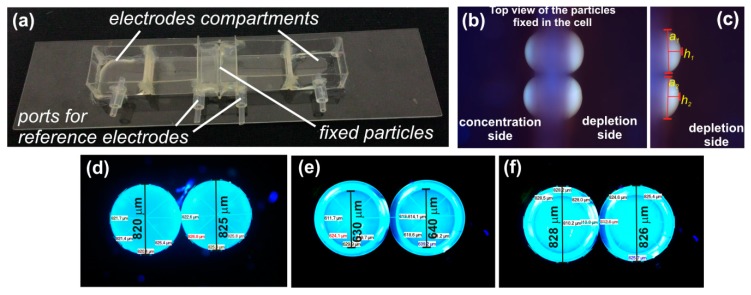
(**a**) Picture of the electrochemical cell used in the experiments; (**b**) picture of a two-particle system as seen under a microscope after it was fixed in the electrochemical cell and the particles were fully swollen; (**c**) detail of the swollen two-particle system on its depletion side. The depicted distances show how the surface areas used in calculating the current density were evaluated. The pictures (**d**), (**e**), and (**f**) show the top view of the two cation-exchange particles used in the preparation of the two-particle membrane system. Figure (**f**) depicts the two resin particles in the dry state before their embedding into the acrylic resin, figures (**e**) and (**f**) show dry resin particles after their embedding into the acrylic resin with corresponding measured distances.

The details about the fabrication of our custom-made electrochemical cell and preparation of the ion-exchange particle based membranes can be found in our previous papers [8,36]. Here, we only outlined the fabrication procedure. The electrochemical cell was designed as a four-chamber cell in which two outer chambers serve for imposing a source signal on the studied system and two inner cells house reference electrodes for the measurement of the electric potential differences occurring on the measured system. The two inner cells were separated with the studied membrane (see detail in Figure 7b). At the same time, the studied membrane was fixed in the system in such a way that it allowed one to observe the interface of the studied particles and the surrounding electrolyte e.g., by using fluorescence microscopy. The cell itself was made of polycarbonate foil and was glued together with the use of UV curable glue Acrifix 182. The cell prepared for measurement is depicted in Figure 7a.

The particle membranes were prepared by the method developed previously [36]. First, resin particles of roughly the same size were selected from a stock (see Figure 7d). The particles were checked for any cracks. A given number of particles were placed in a mold for their encapsulation in a UV curable resin. The particles were placed close to each other (they were touching) and the upper part of the mold was put in place so that the particles did not move during the encapsulation. UV curable resin Acrifix 182 was slowly injected into the mold. Special care was taken not to trap any bubbles and to firmly encapsulate all the particles. Due to the bottom and top cover of the mold the opposing poles of the particles remained free of any glue. The resin was cured under UV exposure source. The particle membrane was recovered from the mold and checked for its quality under a microscope. The top view of the embedded particles in the dry state can be seen in Figure 7e,f. Such a membrane was fixed in the electrochemical cell with the UV curable glue in such a way that (i) the chambers adjacent to the particle membrane were completely separated (no leaks) and (ii) the particles were easily observable under a microscope (see Figure 7b).

### 4.3. Ion-Exchange Surface Area

The active ion-exchange surface area of our membranes is determined from the microscopic images of the resin particles. An example of the surface area determination is in Figure 7c. The particles making up the membrane were geometrically characterized when fully swollen in the used solution. We measured the height and the base of the particle caps that were exposed to the surrounding electrolyte. By determining these geometrical parameters, one could calculate the surface of each particle cap as A_i_ = **π**(*a_i_*^2^/4 + *h_i_*^2^). The overall surface of every membrane was then given as the sum of surfaces of all caps.

### 4.4. Experimental Set-Up

All the electrochemical measurements were performed on galvanostat/potentiostat Gamry 600 in a four-electrode set-up. Two gold wires were used as source electrodes (current circuit) and these were placed in the outer chambers of the cell. Two reference silver/silver chloride electrodes were used as micro-reference electrodes. Their distance from the membrane under study was 6 mm on either side of the membrane. The electrochemical cell was placed under a microscope Olympus BX51WI with an attached color camera Pixelink PL-D775CU. The electrochemical experiments were controlled by software Gamry Framework, the camera by PixeLink Capture OEM.

### 4.5. Measurements

The current–voltage curve was measured by setting a given current load and measuring the voltage response of the system. Specifically the current load started at 0 μA and was stepped up at a rate of 1 μA/s until a given final load was reached. The final load was set in such a way that the length of the overlimiting region was roughly of the same length as the length of the underlimiting region. These values strongly depended on the ion-exchange surface of the given membranes. These experiments were carried out with KCl solution of concentration of 0.01 M with addition of fluorescein at its final concentration of 10^−5^ M. When running the polarization curve, we also imaged the interface between the membrane and the electrolyte solution on its depletion side. The images were taken so that we obtained a characteristics image for each applied current.

The experiments aimed at determining the pH changes associated with a possible water splitting reaction were performed by running chronoamperometric measurements under a given voltage. The duration of these experiments was 5 min. After five minutes, the electrolytes in all four chambers were thoroughly mixed and their pH was measured. In these experiments we used 0.001 M KCl in the chambers adjacent to the membrane and 1M KCl in the source compartments. In the case of the measurement on the anion-exchange particle system we also added another compartment with a phosphate buffer to limit the possible effect of the electrode reaction on pH changes in the system.

## 5. Conclusions

Performed experiments with cation-exchange particle membranes of an increasing number of ion-exchange domain showed that (i) there was no significant effect of the increasing number of particles on the current–voltage curves, (ii) the main mechanism of the overlimiting current for the studied cation-exchange particle systems was electroconvection, which manifests itself very similarly in all cases, and (iii) water splitting did not contribute to the overlimiting current in the case of the cation-exchange particle systems unlike the anion-exchange particle, which showed pronounced susceptibility to water splitting. Quantitative differences in the behavior of the studied membranes were observed, e.g., different values of the limiting current density. These differences were mainly caused by the way the particles were incorporated into the membrane (mutual position, their available surface) rather than the number of the individual particles.

## Figures and Tables

**Table 1:** Characteristics of the individual studied systems and their ion-exchange area.

Membrane	Particle Surfaces	Overall Surface/mm^2^	Limiting Current Density/A/m^2^	Slope of Underlimiting Region/A/m^2^/V	Slope of Overlimiting Region/A/m^2^/V
One-particle membrane	S_1_ = 0.52 mm^2^	0.52	38.4	72.7	60.5
Two-particle membrane	S_1_ = 0.63 mm^2^ S_2_ = 0.67 mm^2^	1.3	31.4	73	43.6
Three-particle membrane	S_1_ = 0.49 mm^2^ S_2_ = 0.50 mm^2^ S_3_ = 0.51 mm^2^	1.5	34.7	41	33.2
Four-particle membrane	S_1_ = 0.29 mm^2^ S_2_ = 0.20 mm^2^ S_3_ = 0.26 mm^2^ S_3_ = 0.28 mm^2^	1.03	40	64.4	44.6

## Abbreviations

CVC	Current–voltage curve
DC	Direct current
